# The Relationship Between COVID-19 Infection Rates and Social Determinants of Health in Broward and Miami-Dade Counties, Florida

**DOI:** 10.7759/cureus.17524

**Published:** 2021-08-28

**Authors:** Lindsey A Taylor, Jarrod Sheehan, Ariel Paz, Monica Tromer, Erica Pieper, Iman Squires, Aysha Nuhuman, Radleigh Santos, Robin J Jacobs

**Affiliations:** 1 Internal Medicine, Nova Southeastern University Dr. Kiran C. Patel College of Osteopathic Medicine, Fort Lauderdale, USA; 2 Statistics, Nova Southeastern University, Fort Lauderdale, USA; 3 Medical and Behavioral Research, Health Informatics, Medical Education, Nova Southeastern University, Fort Lauderdale, USA

**Keywords:** covid-19, socio-economic factors, race inequities, per capita income, florida

## Abstract

Objective

To determine the relationship between per capita income and COVID-19 cases in Broward and Miami-Dade Counties of Florida, USA.

Background

Low socioeconomic status predisposes individuals to worse health outcomes. For example, during the 2003 SARS-CoV pandemic and the 2009 H1N1 influenza pandemic disadvantaged individuals were more likely to become infected. More recently, a study found that deaths due to COVID-19 were associated with disadvantaged areas across the United States. South Florida, in particular Broward and Miami-Dade Counties, has experienced a significant burden of coronavirus cases. Investigating the association of income on coronavirus cases in Broward and Miami-Dade Counties may aid in identifying and treating those individuals at increased risk.

Methods

This retrospective cross-sectional study used data gathered by the Florida Department of Health and 2018 U.S. Census. COVID-19 cases from March 2 - November 1, 2020 were tallied by ZIP code in Florida’s Broward and Miami-Dade Counties and scaled per housing unit. An exhaustive regression analysis using County “Miami-Dade” or “Broward,” sex, race, ethnicity, median age, and estimated per capita income was performed for each combination of independent variables in MATLAB (MathWorks, Natick, USA). Regression models were evaluated using both adjusted R-squared and the Akaike Information Criterion, along with the number of significant predictors. The most optimal model with the highest number of significant predictors was selected.

Results

Among all other variables, sex, race, and ethnicity as the variables that best predicted COVID-19 cases per housing unit within a certain ZIP code. The adjusted R-squared of this optimal model was 0.5062, indicating that within each ZIP code in Broward and Miami-Dade Counties 50.62% of the variance in COVID-19 cases per housing unit can be explained by these variables. A significant relationship was found between the number of COVID-19 cases and individuals who were Black or African American (*p* < 0.001), individuals who were Hispanic or Latino (*p* < 0.001), and male to female ratio (*p* = 0.016). Per capita income, age, and county were not statistically significant predictors in any model tested.

Conclusions

Racial and gender disparities may be more significant contributors to COVID-19 cases than per capita income in housing units. Based on the results of this study, investigators may consider applying this model to similar variables in order to inform the management and prevention of cases in the present and future pandemics.

## Introduction

The inequities faced by those of lower socioeconomic status within the current COVID-19 pandemic elucidate the vulnerabilities of such communities when a public health crisis threatens an area. Individuals with low income are predisposed to worse health outcomes because the existing socioeconomic gap results in inequities in social determinants of health, including access to health care services and quality of education [1]. Additionally, those with chronic health conditions are more likely to experience income inequity, leaving these populations more vulnerable to illnesses that prey on individuals with co-morbid conditions [2]. Through analysis of what makes an individual more susceptible to illness, a more informed approach to the distribution of funds can be employed by public health officials in future pandemics.

Previous disease outbreaks and low socioeconomic status

Individuals with a low income have historically experienced disproportionately negative health consequences during infectious disease outbreaks. During the influenza seasons of 2010 and 2011, the incidence of influenza-related hospitalization increased in census tract areas with greater levels of poverty [3]. “Possible contributing factors [to this higher incidence] are lower vaccination rates in residents of poorer census tracts, poverty-related crowding with higher rates of influenza transmission, and higher prevalence of medical conditions predisposing persons to influenza complications in poorer areas” [3]. 

In 2009, the H1N1 novel influenza virus affected over 60 million people and resulted in 12,000 deaths. Lowcock, et al. found that one was more likely to be infected and subsequently hospitalized due to H1N1 if he or she lived in materially deprived neighborhoods, neighborhoods with high total deprivation, or areas in the lowest employment quintile [4]. Despite accounting for the contribution of other factors such as obesity, smoking, and family doctor use, Lowcock, et al. found that the biggest contribution to the risk of hospitalization and infection involved poor social determinants of health and socioeconomic status [4]. For example, having a high school education or less and living in a neighborhood with high material or total deprivation were associated with higher levels of hospitalization in the first phase of the H1N1 2009 pandemic [4]. 

The study pertaining to the initial severe acute respiratory syndrome coronavirus (SARS-CoV) pandemic of 2003 also found that poorer socioeconomic factors contributed to increased infection rates. SARS-CoV originated in China and infected over 8,000 people, ultimately claiming over 700 lives. A study conducted by Bucchianeri noted that regardless of the measure used for socioeconomic status (SES), whether it be income, education, or occupation, a correlation between SES and risk of infection existed [5].

The scholarly works above demonstrate the deadly role lower socioeconomic status plays in infection during previous disease outbreaks. Their observations establish the need to investigate how socioeconomic status correlates with the current COVID-19 pandemic.

Race and health status

In addition to income status, race and ethnicity also greatly impact infection rates. Certain diseases and infection rates have been shown to be higher in minority populations such as Hispanics and African Americans. Ethnic groups often reside closely together in communities, a factor that can contribute to higher infection rates within a population [6]. For example, the higher rates of *Helicobacter pylori*, *Cytomegalovirus*, herpes simplex virus- 1 (HSV-1), and hepatitis B virus (HBV) amongst African American and Mexican-born Americans have been partly attributed to the proximity of living quarters which may facilitate the spread of contagion [7]. Zajacova, et al. also found that discrimination among certain ethnic groups aside from income can cause significant stress that may further lower the immune system, therefore predisposing individuals to infection [7]. Furthermore, a study conducted by Veterans Affairs also revealed that COVID-19 infection rates among African Americans were higher, but this was not proportionally reported due to less media coverage and epidemiological investigations aimed at the African American community [8]. Rentsch, et al. found that Black and Hispanic individuals experience an excess burden of SARS-CoV-2 infection that cannot be entirely explained by these individual co-morbid conditions or where they reside or receive healthcare [8]. To explain this increase in COVID-19 burden, Adegunsoye, et al. found that COVID-19 infections were higher in the African American population due to crowded home settings and overrepresentation of the African American population in lower-wage public service occupations [9]. These studies indicate that infection rates are notably increased among certain racial/ethnic groups. Though most of the studies do not focus solely on income level, it is shown that increased infection rates, regardless of illness, are prevalent within non-Caucasian demographics due to a multitude of socioeconomic factors. 

Social determinants of health and COVID-19 

Many studies research how race and age demographics affect individuals but fail to look at an individual’s social position as a contributing factor for their health status [8-9]. To investigate socioeconomic status further, a study conducted in a Massachusetts hospital by Cromer, et al. (2020) investigated individuals who tested positive for COVID-19 by census tract and survey to look for a link between COVID-19 and other economic identifiers. This study found that those who were of the male sex, had a preferred language other than English, had Medicaid, or were uninsured, and a household number of greater than five had a greater infection rate [10]. Although these were not direct indicators of income, an individual who meets more than one of these factors is at greater risk of being infected. Thus, these factors should be investigated as further variables to indicate vulnerability. 

Poverty has been seen to play a major role in increased infection rate with COVID-19. Several factors increase the exposure risk of those with lower SES to COVID-19. Poor housing conditions, limited access to personal outdoor space, and overcrowding will reduce compliance with social distancing. Moreover, people who have a financial disadvantage are often employed in occupations that do not provide opportunities to work from home [11]. This lack of access, along with unstable incomes exacerbated by the COVID-19 pandemic, places those of lower SES at a disadvantage. 

These disadvantages faced by minorities are not unique to the geographic area of study. Patel, et al. referenced United Kingdom statistics, but the trend of close quarters causing increased infection rates was also seen in the high incidence of COVID-19 cases in New York City and Miami, United States [11]. An American study found that “. . . a larger number of deaths was associated with a larger percent of county residents living in poverty, living in deep poverty, a higher incidence of low weight births, and with the county being designated as urban” [12]. Impoverished areas were more affected in the early weeks of the pandemic with an ultimate shift to higher infection rates within higher socioeconomic status communities as time progressed [12]. Ramirez and Lee’s investigation of COVID-19 in Colorado, United States also found a pattern of high COVID-19 infection rates in poorer communities [13]. “Social and health determinants associated with higher COVID-19-related deaths were population density and asthma, indicative of urban areas, and poverty and unemployment, suggestive of rural areas” [13]. The social, health and psychological burdens of COVID-19 pose an increased risk to economically vulnerable populations. By elucidating the socioeconomic factors that contribute to the increased burden within this public health crisis, improved prevention and detection strategies can be implemented in these highly susceptible areas.

South Florida and COVID-19

Broward and Miami-Dade Counties are two counties heavily burdened with COVID-19 infection. As of March 27, 2021, there were 208,777 and 434,525 reported positive cases of COVID-19 of Florida residents in Broward and Miami-Dade Counties, respectively [14]. The most recent estimated population of Broward County and Miami-Dade County, Florida is 1,926,205 and 2,699,428 people, respectively [15]. The combined population is 4,625,623. The population composition in Broward and Miami-Dade Counties, respectively, is 30.2% and 17.7% Black or African American alone, as well as 63.1% and 79.0% White alone [14,15]. 31.1% and 69.4% are Hispanic or Latino, while 34.8% and 12.9% are White alone, not Hispanic or Latino in Broward and Miami-Dade Counties [14-15]. In addition, a significant portion of both counties is suffering from poverty. According to 2019 United States Census Bureau estimates, Miami-Dade and Broward Counties have a poverty rate of 17.1% and 13.1% respectively [15]. Based on the data from Lowcock et al. as well as Bucchianeri describing the 2011 H1N1 and SARS-CoV 2003 health crises, lower income individuals had increased infection rates and subsequently worse health outcomes [4-5]. In addition, deaths due to COVID-19 are linked with poorer areas across the United States [12]. Considering the findings of previous novel health crises as well as the national findings of the current pandemic, this investigation delved into different socioeconomic factors’ effects on the coronavirus infectivity rates within Broward and Miami-Dade Counties. Our study, thus, seeks to identify the presence of a relationship between COVID-19 infectivity rate and per capita income, race, gender, age, ethnicity, and county tallied by ZIP code and scaled per housing unit.

## Materials and methods

**Figure 1:**
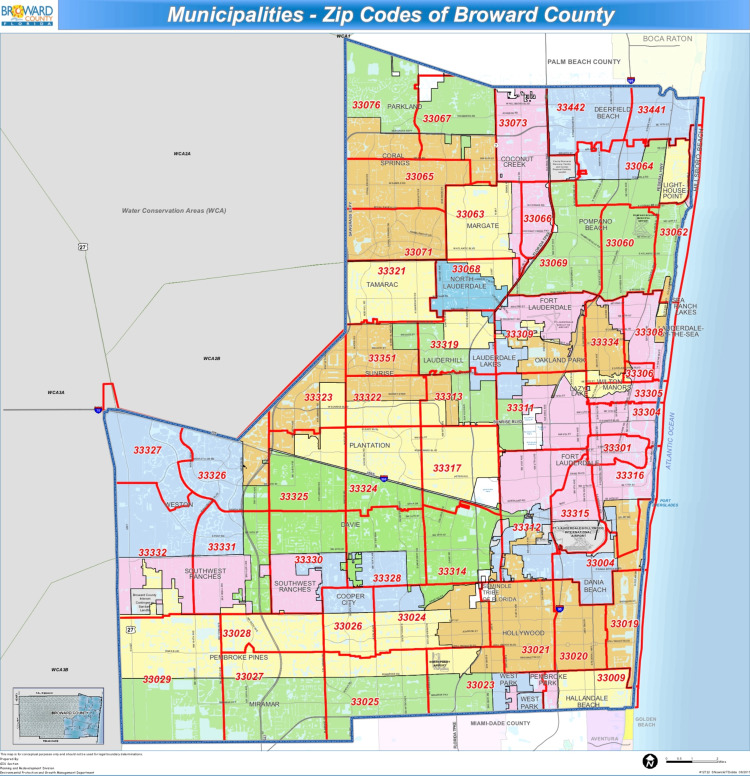
Municipalities – ZIP Codes of Broward County

**Figure 2:**
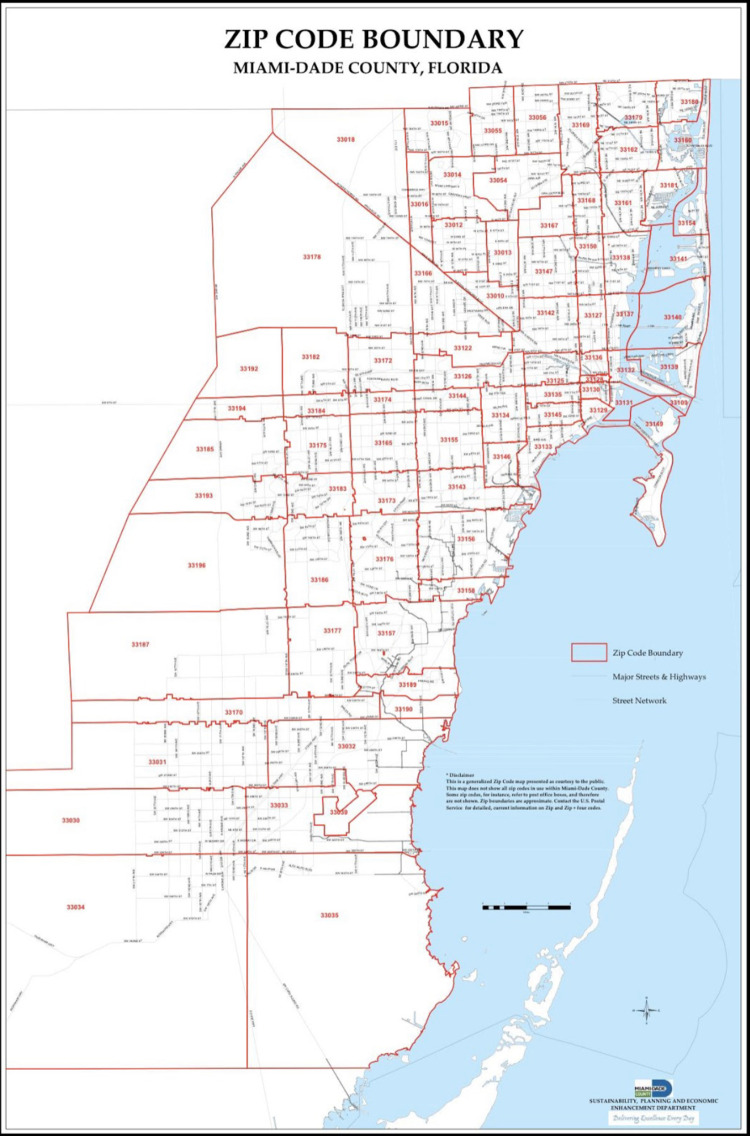
ZIP Code Boundary of Miami-Dade County

This retrospective cross-sectional study utilized observational data to potentially establish a relationship between COVID-19 and social determinants of health. The target community population was all persons testing positive for COVID-19 reported by the state of Florida per ZIP code within Broward and Miami-Dade Counties as demonstrated in Figures 1, 2 [16-17]. 

The intended study sample is based off those documented in public databases provided by the Florida Department of Health (FDoH) and the United States Census Bureau. Relevant demographics needed to increase the validity of the study were income statistics, poverty level, households with an area, age, gender, and race, all collected from the 2018 American Community Survey within the United States Census Bureau database. Miami-Dade and Broward Counties were chosen based on their diverse population, as well as the high density of people within a smaller area. Together, there are a total of 1.6 million households within this area making it a diverse location to investigate COVID-19 infection rates. The median household income for Broward County and Miami-Dade County is $59,547 and $51,347 with 13.1% and 17.1% in poverty, respectively [18-19]. This level of poverty is significantly higher than the national poverty rate of 10.5% [15]. The proportion infected with COVID-19 within the population for both counties was 13.9% [14]. Ultimately, this area allowed for a more in-depth, unique analysis of COVID-19 infection rates, socioeconomic status, and other social determinants of health. 

Data available only within ZIP code format from the Florida Department of Health were collected between March 2, 2020 through November 1, 2020. To ascertain the number of confirmed COVID-19 cases within certain ZIP codes of each area, this set of data was parsed to only include the COVID-19 cases accumulated within all ZIP codes in Broward and Miami-Dade Counties in Florida. The socioeconomic data were obtained from the 2018 American Community Survey provided by the U.S. Census Bureau also parsed by ZIP code boundaries. After the data had been collected, the instrument chosen to detail goodness-of-fit of multivariate regression models was the Akaike’s Information Criterion (AIC) in exploratory factor analysis (EFA). This criterion is used in large models which balance sensitivity and specificity by choosing the model that has the best penalized log-likelihood [20]. Using MATLAB (MathWorks, Natick, USA), a multivariate regression was done by assessing average per capita income level, COVID-19 infection rate, race, ethnicity, age, county, and male to female ratio tallied by ZIP code and scaled per housing unit. Then, using the AIC, the model with the highest goodness of fit was chosen. The information was then scaled per housing unit and per individual. The final data included per capita income, male to female ratio, race, ethnicity, age, county, and number of households within every ZCTA (ZIP Code Tabulation Area) in Broward and Miami-Dade Counties.

## Results

**Figure 3:**
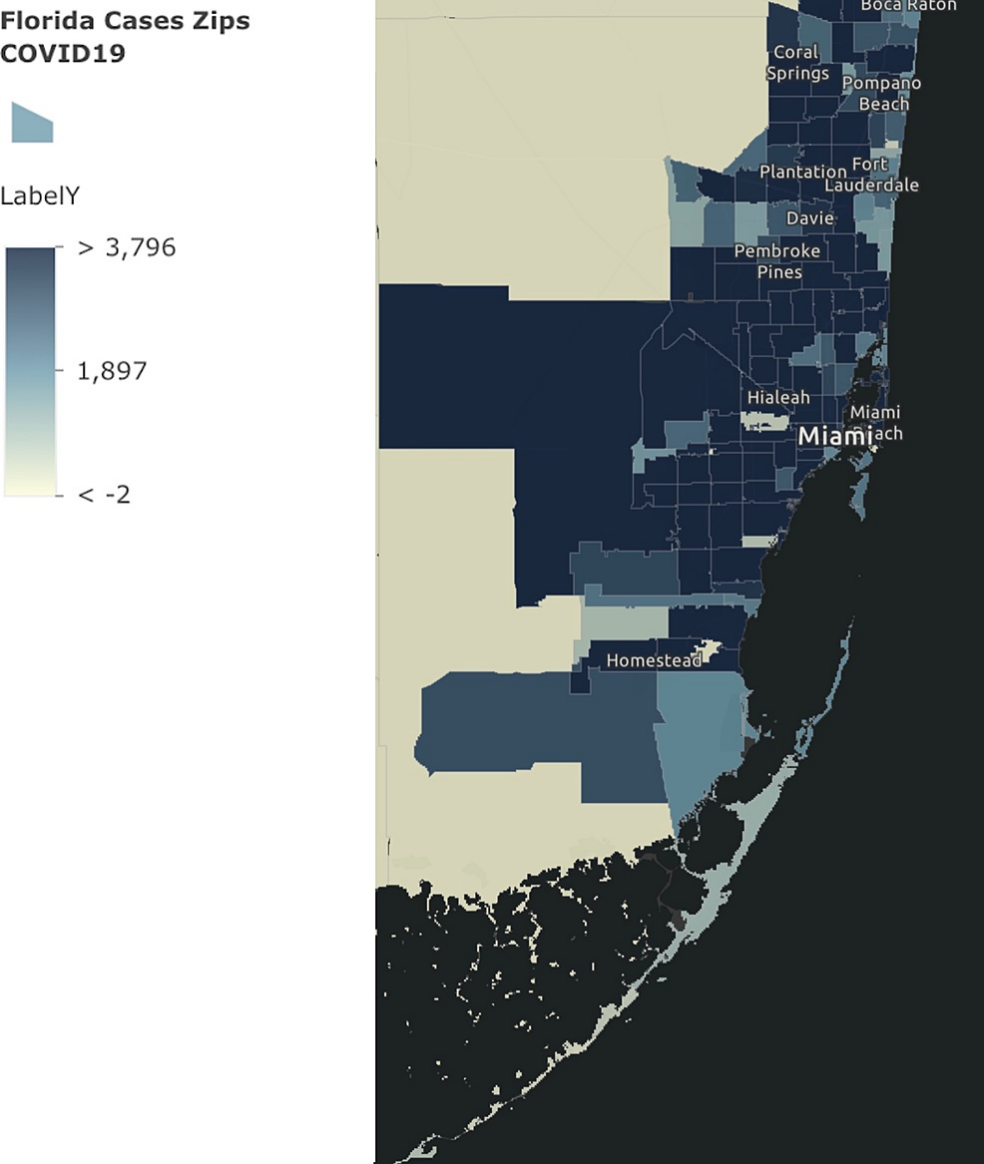
Florida Department of Health ArcGIS Reconstruction of COVID-19 Cases in Broward and Miami-Dade Counties, Florida LabelY is COVID-19 cases recorded to date. ArcGIS is developed by Environmental Systems Research Institute, Redlands, USA.

A multivariate regression was used to analyze 130 ZIP codes within Broward and Miami-Dade Counties, Florida with COVID-19 cases from March 1st to November 2nd, 2020. The COVID-19 case distribution by ZIP code is illustrated in Figure 3 [14].

Using the legend, it is found that a majority of the counties had more than 4,000 cases within each section further solidifying the high burden of COVID-19 cases present within the two counties tested. The final regression model chosen was based on the optimization of Akaike’s Informational Criterion and was least likely to result in sample prediction error. The optimal regression model chosen included sex, race, and ethnicity as the variables that best predicted COVID-19 cases per housing unit within a certain ZIP code. The chosen model was: 

COVID19 Cases = -0.1005 + 0.0545 (Black or African American) + 0.0793 (Hispanic or Latino) + 0.1222 (Male to Female Ratio).

The adjusted R-squared of this model was 0.5062, indicating that within each ZIP code in Broward and Miami-Dade Counties 50.62% of the variance in COVID-19 cases per housing unit can be explained by these three variables. As seen below in Table 1, the standard of error for the model was 0.0727 suggesting this was a model with increased accuracy. 

**Table 1:** Regression Summary Output for Per Household COVID-19 Cases Analysis

SUMMARY OUTPUT						
Regression Statistics						
Multiple R	0.7195					
R Square	0.5176					
Adjusted R Square	0.5062					
Standard Error	0.0727					
Observations	131					
Analysis of Variance						
	Degrees of Freedom	Sum of Squares	Mean of Squares	F ratio	Significance F ratio	
Regression	3	0.7209	0.2403	45.4284	0.0000	
Residual	127	0.6718	0.0053			
Total	130	1.3927				
	Coefficients	Standard Error	t Statistic	P-value	Lower 95%	Upper 95%
Intercept	-0.1005	0.0532	-1.8897	0.0611	-0.2057	0.0047
Black or African American	0.0545	0.0104	5.2527	0.0000	0.0339	0.0750
Hispanic or Latino (Total Population)	0.0793	0.0069	11.4549	0.0000	0.0656	0.0930
Male to Female Ratio	0.1222	0.0501	2.4370	0.0162	0.0230	0.2213

In the model chosen, a significant relationship was found between the number of COVID-19 cases and individuals who were Black or African American (p < 0.001), individuals who were Hispanic or Latino (p < 0.001), and male to female ratio (*p* = 0.016). In Table 1, an increase in one Black or African American individual per 1,000 total households resulted in an expected increase of 54.50 cases of COVID-19 per 1 million households, with a 95% C.I. of (33.90, 75.00). An increase in one Hispanic or Latino individual per 1,000 total households resulted in an expected increase of 79.30 cases of COVID-19 per 1 million households, with a 95% C.I. of (65.60, 93.00). An increase in 0.1 of male-to-female ratio resulted in an expected increase of 122.20 cases of COVID-19 per 1 million households, with a 95% C.I. of (23.00, 221.30). By looking at the obtained values as per 1,000 actual people in the population, the predictor variables are the relative amount of each race in a given ZIP code, rather than counts, so the issue of “double counting” becomes obsolete. Per capita income, age, White race and county were not statistically significant predictors in any model tested; thus, they were not included in the optimal regression. Finally, only 130 ZIP codes of the 131 were tested due to one ZIP code having more COVID-19 cases than people that resided in the ZIP code. This discrepancy eliminated this area from being included in the model. 

## Discussion

A significant relationship between the number of COVID-19 cases and individuals who were Black or African American, individuals who were Hispanic or Latino, and households with a higher male to female ratio was found. Per capita income, age, White race, and county were not statistically significant predictors in any model tested. It is important to note that this study did not account for individuals who contracted COVID-19 and were not tested, those not tested at state-supported sites, and those who received false-negative test results. Therefore, the trends highlighted in this dataset are likely underestimated. Additionally, combination races were excluded to decrease the likelihood these variables were counted twice. A much larger variation in Hispanic or Latino density per ZIP code versus White was found possibly contributing to the significance of Hispanic or Latino as a predictor rather than White. The final results indicate that racial and gender disparities were more significant contributors to COVID-19 cases than per capita income when scaled per housing unit. Identifying populations at an increased risk of contracting COVID-19 may aid public health officials in elucidating specific psychosocial factors that make these populations more vulnerable than others.

Per capita income did not play a role in COVID-19 cases within the Broward and Miami-Dade Counties, but this does not exclude income as an important underlying factor in other communities. There remains a need for interventions to decouple income and health, or to reduce inequalities in income so that an “emergence of a 21st century health-poverty trap and the further widening and hardening of socioeconomic inequalities in health” can be prevented [1]. Despite this need, many are still unable to receive the care that they need when resources are limited. Future research should investigate per capita income and chronic disease burden in COVID-19 hotspot areas to ascertain their effect on contraction of this infectious disease so that efforts can be made toward health equity. “Furthermore, a spatial overlap of high rates of chronic diseases with high rates of COVID-19 may suggest a broader syndemic health burden, where comorbidities intersect with inequality of social determinants of health” [13]. This study highlights the multifactorial nature of COVID-19 infectivity; thus, research efforts should be focused on the intersection of a multitude of factors. A better understanding of what increases the infectivity of coronavirus would especially benefit an area with a significant amount of burden, as well as a rate of poverty higher than the national average [15,18-19].

This study can be applied in public health and clinical settings. This model highlights the vulnerability of diverse communities with greater diversity and with an increase in male populations display more vulnerability within Broward and Miami-Dade Counties. By applying this model epidemiologically, public health officials may implement specific prevention strategies for the most vulnerable populations to decrease the overall number of cases in the present and future pandemics. Public health prevention efforts can be focused on communities that have an increased risk so that education can improve prevention efforts. Additionally, clinicians may use this data to inform their patients of demographic risk factors for contracting COVID-19. Through education about risk and prevention strategies, patients can better protect themselves and their families from this disease. Efforts to target vulnerable populations with additional resources can work toward decreasing health disparities.

Limitations and future research

Due to the nature of the design of this study, there was an inability to extrapolate cause and effect. This study merely sheds light on the higher burden of disease found in the minority population of Broward and Miami-Dade Counties. A future area of study should delve into why these areas are more affected by COVID-19 and what public health measures can be taken to correct these injustices. This study highlights the need for public health resources to be focused on diverse communities that have been detrimentally affected by COVID-19. 

## Conclusions

In conclusion, a need for research on COVID-19’s impact on a variety of social determinants of health has been established. This research investigated per capita income, race, ethnicity, age, county, and gender to uncover the variables’ relationship to infection rates within Broward and Miami-Dade Counties, Florida. Race, ethnicity, and gender were significant factors in increased disease contraction per household. Public health efforts should focus on populations that are most vulnerable in order to increase access to care and improve health equity.
